# Cross-cultural adaptation and validation of the theory of Planned Behavior-Hearing Help Seeking: A methodological study

**DOI:** 10.1016/j.bjorl.2025.101660

**Published:** 2025-06-25

**Authors:** Yuxuan He, Menghui Deng, Wei Li, Jia Liu, Xiaomei Chen, Yanni Yang

**Affiliations:** a Army Medical University, School of Nursing, Chongqing, China; b Nursing department, Chengdu Wenjiang District People's Hospital, Chengdu, China

**Keywords:** Hearing help seeking Scale, Chinese version, Hearing loss

## Abstract

**Objective:**

Seeking help for hearing loss is a complex psychological reaction. The purpose of this investigation was to cross-culturally adapt and validate the Theory of Planned Behavior-Hearing Help Seeking (TPB-HHS) to Chinese language and culture.

**Methods:**

A total of 184 middle-aged and older Chinese adults completed the TPB-HHS. The study explored the factor structure and psychometric properties of the Chinese TPB-HHS, including model fit, convergent validity, discriminant validity, internal consistency and retest reliability.

**Results:**

Four factors were identified, explaining 65% of the variance, with significant internal consistency and model fitting. In addition, they had satisfactory retest reliability after one week. In addition, the TPB-HHS scale showed substantial convergent and discriminant validity.

**Conclusion:**

The Chinese TPB-HHS version has been shown to be a reliable and effective tool for measuring hearing test intention in initial community hearing screening. It enables healthcare providers to determine the level of intent to seek help in patients with hearing loss, laying the foundation for interventions to help these patients achieve optimal physical, psychological, and social outcomes.

**Level of evidence:**

2.

## Highlights

• The TPB-HHS is suitable for assessing help-seeking behavior in the community.
• TPB-HHS was cross-culturally adapted to the Chinese language in China.
• The Chinese version TPB-HHS scale items and structure are similar.
• TPB-HHS can be used for assessment, follow-up, and intervention.
• TPB-HHS translates and adapts well to Chinese.

## Introduction

Hearing loss is a prevalent long-lasting health issue in adults characterized by a decrease in hearing ability caused by numerous factors.^1^ Approximately 1.5 billion people worldwide, which accounts for nearly 20% of the global population, have hearing impairments.^2^ Hearing impairment has several negative outcomes such as loneliness, anxiety, depression, social isolation, relationship strain, and an increased likelihood of acquiring dementia.^3^ The rising number of individuals with undetected and untreated hearing impairment leads to a higher demand for healthcare services, resulting in increased economic and social burdens.^4^ Consequently, addressing hearing issues is considered a primary concern in public health. Seeking expert assistance is a typical method for managing hearing issues, however this approach evolves with hearing rehabilitation. The traditional belief that “hearing loss is a natural part of aging and does not need special attention”^5^ has led to an average delay of 8.9 years before seeking professional help.^6^ Delaying the initial care-seeking procedure might make treatment more difficult and raise the risk of long-term consequences from not receiving early intervention.^7^ A significant percentage of middle-aged and older adults have been forced to deal with unresolved hearing impairment and suffer from its negative effects for years or even decades. Untreated hearing loss not only impairs a person's physical and mental well-being, but it also puts them under financial strain, increases social isolation, and drastically lowers their quality of life.^8^ The treatment process becomes much more complex when patients' hearing loss gradually worsens and they run the danger of developing complications including dementia^9^, ^10^ and depression^11^ which call for more care. Unfortunately, many of the potential advantages that may have been attained with early intervention are lost as a result of delayed therapy.^12^ Poor healthcare access and significant doctor-patient communication barriers can also contribute to a higher likelihood of delayed access to medical care.^13^ Early identification and assessment of a hearing problem's help-seeking stage are crucial for understanding this population and creating individualized interventions.

Several questionnaires have been developed to evaluate various framework constructs, such as the University of Rhode Island Change Assessment (URICA) for analyzing the structure of the Cross Theoretical Stages Model^14^ and the Hearing Beliefs Questionnaire for the Health Beliefs Model (HBM).^15^ However, both questionnaires have limitations in assessing help-seeking behaviors. URICA can identify persons in advanced stages of the process who are more inclined to use hearing aids, but it cannot predict those who will continue using hearing interventions in the long run.^16^ The HBM fails to consider adherence, which reduces the accuracy of explanations for long-term behavior change.^17^ Hence, a more practical, straightforward, and precise hearing help seeking assessment approach is required for early detection and evaluation.

Arnold and colleagues^18^ created the Theory of Planned Behavior-based Hearing Help Seeking Questionnaire (TPB-HHS) to evaluate the factors that impact hearing help-seeking behavior. The questionnaire comprises four subscales: “Attitude” assesses the individual's perception of the hearing test, “Subjective Behavioral Norms” gauges other people's perceptions of the hearing test behavior, and “Perceived Behavioral Control” evaluates the individual's confidence in their ability to perform the hearing test behavior. Participants with high scores on Attitude, Subjective Behavioral Norms, and Perceived Behavioral Control questions showed a strong inclination to seek help, which influenced their actual behavior of seeking help for hearing issues. Thus, these four subscales can be viewed as indicators of the probability of engaging in hearing test activity, including internal elements like attitudes and behavioral control, as well as external factors such as subjective norms.^19^

The reliability and validity of the TPB-HHS questionnaire have been confirmed in Western populations, but its applicability to Asian populations, particularly Chinese, is uncertain due to potential cultural differences affecting attitudes and behaviors. The structural validity, discriminant validity, and convergent validity of the TPB-HHS scale in psychometric properties remain uncertain. Hence, the aim of this study is in two parts: 1) To translate TPB-HHS into Chinese and modify it for cross-cultural use; 2) Test the reliability (internal consistency and retest reliability) and validity (convergence validity) of TPB-HHS in Chinese samples to enhance the tool's applicability. We think that these discoveries will enhance past research, supplement the current knowledge base, and progress the field of hearing-related research and clinical practice.

## Methods

**Fig. 1:**
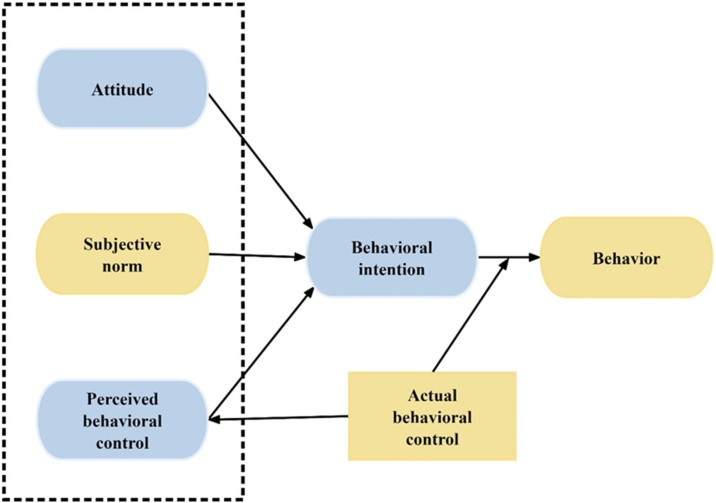
A simple model of the theory of planned behavior (Ajzen1991). Fig. 1 illustrates a basic model of the theory of planned conduct. The approach suggests that intentions are primarily shaped by attitudes, subjective norms, and perceived behavioral control. Greater intention leads to increased likelihood of behavior execution.

This study's methodology is restricted to cross-cultural adaptation and validation. It was approved by the Medical Ethics Committee of Second Affiliated Hospital of Army Medical University, PLA. Each participant gave their approval to participate after signing informed consent forms. The TPB-HHS is an 18-item self-report measure designed to examine the psychological effects of hearing tests on people who have hearing loss. Four dimensions make up the structure: intention, attitude, subjective norms, and perceived behavioral control. Fig. 1 displays the structure of the model. Participants used a visual analog scale to rank the items, higher scores meant that they were more likely to request a hearing test.

In accordance with the internationally recognized American Academy of Orthopedic Surgeons (AAOS) questionnaire adaptation criteria, TPB-HHS was officially authorized for cross-cultural adaptation. The process included the following steps, which were further explained as follows: (1) Forward translation, (2) Translation review, (3) Back translation, (4) Expert letter consultation, and (5) Pre-survey.

1) Two qualified translators, an English teacher and a nurse in the Department of Otolaryngology, who are fluent in English and native speakers of the target language, translated the original questionnaire items, instructions for completing it, and scoring guidelines while maintaining the original questionnaire's meaning.
2) To construct version 1, a nursing professional with study abroad experience and two forward translators worked together to review the Chinese version to ensure that the language expression was accurate and fully adapted to the cultural background and linguistic customs.
3) Version 1 was back-translated into English by a medical doctor of otolaryngology with study abroad experience and a college English major teacher. The two back translators had not communicated with the original questionnaire before the back translation.
4) After careful translation, we assembled an expert group consisting of three otolaryngology professionals, four geriatric nursing specialists, two psychologists, and two head nurses with specific training. The judges assessed the questionnaire's equivalency, readability, and intelligibility. Following a review and revision of the expert team's viewpoints and significance, the new Chinese version (version 2) was finally finished.
5) The adapted version was used to conduct a pilot study in Chinese community-dwelling middle-aged and older individuals aged ≥45-years with subjective hearing loss. People with hearing loss were examined in the survey by asking if they had the condition. Individuals who refused to take part and whose Mini-Mental State Examination (MMSE) score was fell below the educational threshold (the elementary group ≤17-points, the primary group ≤20-points, and the intermediate and above group ≤24-points^20^ were not included.

### Statistical analysis

Statistical analysis was conducted using SPSS 27.0 and Amos 24.0. Before conducting the analysis, carefully inspect and remove any missing data and outliers. Continuous variables will be represented by the mean and Standard Deviation (SD), whereas categorical variables will be shown as frequency and percentage. The structural validity of TPB-HHS was assessed by Exploratory Factor Analysis (EFA) using the maximum variance approach. The factor structure of TPB-HHS was verified using Kaiser–Meyer–Olkin (KMO) measurements and Bartlett sphericity test. The optimal KMO estimate should exceed 0.7, and the Bartlett test shows significance (p < 0.05), suggesting a suitable factor structure. The TPB-HHS scale underwent Confirmatory Factor Analysis (CFA), the model fit was considered satisfactory when the following conditions were fulfilled: χ²/df ranged from 1 to 3, goodness of Fit Index (GFI), Normed Fit Index (NFI), and Comparative Fit Index (CFI) were all greater than 0.8, and root-mean-square error of approximation (RMSEA) was less than 0.08. Composite Reliability (CR), Average Variance Extracted (AVE), and Heterotrait-Monotrait ratio (HTMT) will be calculated to assess the convergence validity and discriminative validity of the scale. If CR is greater than 0.7 and AVE is greater than 0.5, the convergence validity is good. If HTMT is less than 0.9, the discriminative validity is good.^21^ Cronbach's α is computed to evaluate the internal consistency of TPB-HHS, with a threshold of over 0.7 being satisfactory. A Pearson correlation coefficient was established between the baseline TPB-HHS score and the 1-week retest score to assess the retest's reliability.

## Results

### Participant characteristics

A total of 184 eligible middle-aged and older adults were invited to participate in the study during a nine-month recruitment period. Of these, 184 middle-aged and older adults agreed to participate, resulting in an overall response rate of 85.98%. The mean age of participants in the study was 66.95-years (SD = 7.97), and 56.40% of respondents were female. In addition, the respondents had an average of 9.93-years of schooling (SD = 3.24), the duration of hearing loss was 7.66-years (SD = 11.39), and only 16.30% had requested hearing help in the past, as more details are shown in Table 1.

**Table 1:** Participant characteristics (n = 184).

Variables	Mean (SD) or n (%)	Range
Age (year)	66.95 (7.97)	49∼91
Gender		
Male	80 (43.5%)	
Female	104 (56.5%)	
Educational attainment (year)^a^	9.93 (3.24)	0∼23
Physical condition		
Poor	30 (16.30%)	
Fair	107 (58.20%)	
Good	47 (25.50%)	
Receive hearing education^b^	46 (25.00%)	
Hearing loss duration	7.66 (11.39)	1∼64
Hearing help seeking^c^	30 (16.30%)	
MoCA	22.64 (3.57)	0∼30
HHIE	25.03 (14.87)	0∼100
Emotion	12.87 (7.66)	0∼50
Socialization	11.96 (8.62)	0∼50
hearWHO	46.40 (13.99)	0∼100

a Including the total number of years of primary, middle, high school and university education. HHIE.

b Including hearing-related education from television programs, the Internet, doctors and nurses, books and newspapers, community talks or friends and relatives.

c Including consultation with specialist audiologists and nurses or visits to audiology clinics. Hearing Handicap Inventory for Elderly. MoCA, Montreal Cognitive Assessment Scale; hearWHO, a software to measure objective listening.

### Exploratory factor analysis

The TPB-HHS factor structure was examined using principal component analysis and maximum rotation. The high KMO value of 0.888 and the statistically significant Bartlett's sphericity test (χ^2^ = 1858.50, ***p < 0.001) suggest a strong correlation among the variables, making factor analysis suitable. The EFA identified a four-factor structure that accounted for 64.878% of the variation (Factor 1 = 22.00%; Factor 2 = 20.30%; Factor 3 = 13.10%; Factor 4 = 11.60%). Table 2 provides a breakdown of the loadings for each item.

**Table 2:** Factor loading of exploratory factor analysis.

Item nº	Subscale	Factor 1	Factor 2	Factor 3	Factor 4
3	Intention	0.665			
7	Intention	0.746			
8	Intention	0.753			
12	Intention	0.616			
13	Intention	0.791			
16	Intention	0.674			
1	Attitude		0.597		
2	Attitude		0.722		
5	Attitude		0.779		
6	Attitude		0.581		
9	Attitude		0.797		
15	Attitude		0.708		
10	Subjective Norm			0.698	
11	Subjective Norm			0.839	
18	Subjective Norm			0.776	
4	Perceived Behavioral Control				0.845
14	Perceived Behavioral Control				0.706
17	Perceived Behavioral Control				0.713
Eigenvalues		7.83	1.78	1.37	1.08
Percentage of variance		22.01%	20.32%	13.08%	11.58%

Following rotation, the items exhibited moderate to strong loading on the four criteria. Factor 1 included question items 3, 7, 8, 12, 13, and 16; Factor 2 included items 1, 2, 5, 6, 9, and 15; Factor 3 included items 10, 11, and 18; and Factor 4 included things 4, 14, and 17.

### Confirmatory factor analysis

The sample was explored in depth through CFA to replicate the four-factor structure and to make these four factors correlate with each other in the model and the model did not contain correlated errors. The proposed model's Chi-Square statistic was χ^2^/df = 2.155 (***p < 0.001), and the fit indices for the model were RMSEA = 0.079, NFI = 0.856, GFI = 0.862 and CFI = 0.916, indicating a satisfactory model fit.

CFA were conducted in this study for all dimensions, and four constructs of the model were identified: attitudes, intentions, subjective norms, and perceived behavioral control. The loadings of all the constructs ranged from 0.564 to 0.875 and were significant; in addition, their component reliabilities ranged from 0.7 to 0.9, correspondingly, and the mean-variance extractions met the criterion (> 0.5) except for Perceived Behavioral Control, which was slightly lower than 0.5, but was still within the acceptable range,^22^ thus all four constructs had good convergent validity, and the specific results are shown in Table 3.

**Table 3:** Potential project confidence analyzes.

Dimension	Entry	Standard coefficients	Unstandardized coefficients	Standard error	*t*-value	p-value	Squared multiple correlations	Composite reliability	Average variance extracted
Attitude	A1	0.646	1.000				0.646	0.889	0.576
	A2	0.844	1.169	0.125	9.373	<0.001	0.844		
	A5	0.827	0.998	0.108	9.231	<0.001	0.827		
	A6	0.698	0.988	0.120	8.253	<0.001	0.698		
	A9	0.875	1.210	0.122	9.939	<0.001	0.875		
	A15	0.623	0.950	0.128	7.438	<0.001	0.623		
Intention	I3	0.849	1.000				0.849	0.899	0.602
	I7	0.818	1.018	0.075	13.649	<0.001	0.818		
	I8	0.564	0.660	0.082	8.067	<0.001	0.564		
	I12	0.802	0.923	0.071	12.977	<0.001	0.802		
	I13	0.757	0.925	0.078	11.875	<0.001	0.757		
	I16	0.828	1.017	0.073	13.872	<0.001	0.828		
Subjective Norm	S10	0.725	1.000				0.725	0.748	0.501
	S11	0.798	1.065	0.158	6.743	<0.001	0.798		
	S18	0.583	0.788	0.133	5.917	<0.001	0.583		
Perceived Behavioral Control	C4	0.723	1.000				0.723	0.703	0.443
	C14	0.690	0.945	0.155	6.100	<0.001	0.690		
	C17	0.574	0.761	0.122	6.253	<0.001	0.574		

The HTMT method was used to test the discriminant validity among the factors. From the analyses in Table 4, all HTMT values were all below 0.9, which is an indication of excellent discriminant validity of the scale.

**Table 4:** Heterotrait-monotrait ratio analysis.

	Attitude	Intention	Subjective norm	Perceived behavioral control
Attitude	‒			
Intention	0.852	‒		
Subjective Norm	0.515	0.535	‒	
Perceived Behavioral Control	0.547	0.509	0.226	‒

### Reliability of the TPB-HHS

The reliability of the scales, determined by the suggested four-factor structure, was evaluated with data from 184 individuals at the beginning of the study. Internal consistency results for the whole scale and subscales are as follows: total TPB-HHS (Cronbach's α = 0.90), help-seeking intention (Cronbach's α = 0.89), attitude (Cronbach's α = 0.82), perceived behavioral control (Cronbach's α = 0.60), and subjective norms (Cronbach's α = 0.70).

Retest reliability was assessed using data obtained from multiple measurements on 20 subjects, which displayed in Table 5. The retest correlation coefficients for each subscale are as follows: intention *r* = 0.96 (**p < 0.01), attitude *r* = 0.78 (**p < 0.01), perceived behavioral control *r* = 0.94 (**p < 0.01), and subjective norms *r* = 0.57 (**p < 0.01).

**Table 5:** Reliability of the theory of planned behavior-hearing help seeking.

	Items	Reliability internal consistency	Test-retest reliability
Overall TPB-HHS	18	0.902	
Intention	6	0.887	0.969
Attitude	6	0.821	0.775
Subjective Norm	3	0.704	0.571
Perceived Behavioral Control	3	0.604	0.935

*p < 0.05; **p < 0.01.

All subscales showed statistically significant correlations (Table 6). In China, the correlations between the TPB-HHS subscales varied from 0.18 to 0.73, with a median of 0.43.

**Table 6:** Inter-correlations between the four subscales.

	Intention	Attitude	Perceived behavioral control	Subjective norm
Intention	1	0.732^b^	0.368^b^	0.432^b^
Attitude	0.732^b^	1	0.438^b^	0.454^b^
Perceived Behavioral Control	0.368^b^	0.438^b^	1	0.177^a^
Subjective Norm	0.432^b^	0.454^b^	0.177^a^	1

^a^p < 0.05; ^b^ p < 0.01.

## Discussion

Most communities in underdeveloped countries do not undergo routine hearing assessments.^23^ Behavioral change models like COM-B, TTM, and HBM theories used in audiology research focus on voluntary lifestyle behaviors and may not fully apply to involuntary life experiences related to hearing loss.^17^ Hence, it is essential to identify a theoretical, practical, and simple strategy for predicting the listening assistance behavior of community inhabitants. We cross-culturally adapted TPB-HHS following rigorous rules and assessed its psychometric characteristics in Chinese populations. The results indicate that the scale demonstrates acceptable structural validity, convergent validity, and discriminant validity. Furthermore, it demonstrates strong internal consistency and reliability upon retesting. This study is the first to validate the questionnaire in multiple languages, as far as we know. This study's findings may aid in future research and therapeutic practice linked to hearing.

This discovery aligns with the four-factor framework of the HHS-TPB, in line with the original results in the US. The majority of the factor loadings were above 0.60. The structural validity of the TPB-HHS scale was only deemed acceptable. The internal consistency for Perceived Behavioral Control was found to be low, perhaps because there were too few relevant measurement entries. Due to certain entries displaying weak internal consistency, it somewhat impacted the structural validity of the entire scale. The scale demonstrates strong retest reliability. The index and sub-indexes show strong internal consistency, with a score exceeding 0.70, indicating that the TPB-HHS scale is stable. The internal consistency of the Perceived Behavioral Control sub-index was 0.60, lower than the original US study's results (Cronbach's α = 0.70).^18^ The low alpha values in the findings are mostly caused by the limited diversity of the questions in the sample, which weakens the scale's internal consistency. The little internal consistency could be attributed to the limited number of elements (n = 3).

Seeking assistance for hearing issues is an intricate idea shaped by several psychological causes. This measure evaluated the psychological factors linked to seeking care for hearing issues, focusing on attitudes, intentions, subjective standards, and perceived behavioral control. Older persons who do not seek help may exhibit strong relationships between these issues. The relationship between these factors may vary at different stages of seeking care for hearing issues. As individuals seek help, the reasons impacting their decisions shift from external to internal incentives over time.^24^ Subjective norms could be the primary element impacting decision-making for middle-aged and older persons in the early stages of hearing loss. Attitudes become the main factor influencing whether individuals seek care in the advanced stages of hearing loss.

Thus far, there has been no study conducted on obtaining assistance for hearing problems within the Chinese population. This study introduced the TPB-HHS self-report measuring method for hearing help-seeking in middle-aged and elderly individuals with hearing loss to China. The scale has an appropriate number of items and exhibits strong psychometric properties. TPB-HHS focuses more on the development and alteration of help-seeking intention compared to other help-seeking scales, and takes into account all potential factors that could influence help-seeking intention. The Chinese iteration of TPB-HHS shows potential as a tool for evaluating the hearing-seeking behavior of middle-aged and elderly individuals with hearing impairment in the Chinese population.

The study possesses both advantages and constraints. This is the initial study to authenticate TPB-HHS across various languages and cultures, and acculturation is conducted in accordance with stringent rules. However, considering the study's constraints, the results should be approached with care. The scale lacks standardized validation since it cannot be compared to related variables due to the novelty of the hearing help-seeking instrument. Moreover, Individuals who are reluctant to seek help may find certain questions concerning listening for help behavior to be “meaningless”, leading to confusion and perhaps random scoring. This psychological effect may have skewed the outcomes of the study.

## Conclusion

The TPB-HHS scale is reliable and valid for assessing hearing help-seeking behavior in middle-aged and elderly Chinese individuals. The scale provides a simple and rapid method for screening hearing help-seeking in China and displays high validity and sufficient reliability, including internal consistency, discriminant validity and retest reliability. Hearing assistance is crucial for various extra health concerns. Healthcare professionals, such audiologists and nurses, may use the TPB-HHS to evaluate hearing aid-seeking behavior in research and clinical settings. Meanwhile, further Chinese populations need to validate the psychometric features of the TPB-HHS to give better evidence for its wider application.

## ORCID

Yuxuan He: 0009-0002-3005-3421.

Li Wei: 0000-0001-6576-8836.

Jia Liu: 0009-0001-5697-5769.

Xiaomei Chen: 0000-0001-9822-7067.

Yanni Yang: 0000-0003-4383-5668.

## Funding

This study was supported by a grant from the National Social Science Foundation of China (nº 20BRK039).

## Declaration of competing interest

The authors declare no conflicts of interest.

## References

[1] World Health O (2021).

[2] Haile L.M., Kamenov K., Briant P.S., GBD 2019 Hearing Loss Collaborators (2021). Hearing loss prevalence and years lived with disability, 1990–2019: findings from the Global Burden of Disease Study 2019. Lancet.

[3] Saunders G.H., Chisolm T.H., Wallhagen M.I. (2012). Older adults and hearing help-seeking behaviors. Am J Audiol..

[4] McDaid D., Park A.-L., Chadha S. (2021). Estimating the global costs of hearing loss. International J Audiol.

[5] Humphrey C., Herbst K.G., Faurqi S. (1981). Some characteristics of the hearing-impaired elderly who do not present themselves for rehabilitation. Br J Audiol..

[6] Simpson A.N., Matthews L.J., Cassarly C., Dubno J.R. (2019). Time from hearing-aid candidacy to hearing-aid adoption: a longitudinal cohort study. Ear Hear..

[7] Schönborn D., Asmail F.M., De Sousa K.C. (2020). Characteristics and help-seeking behavior of people failing a smart device self-test for hearing. Am J Audiol..

[8] Cunningham L.L., Tucci D.L. (2017). Hearing loss in adults. N Engl J Med..

[9] Huang A.R., Jiang K., Lin F.R., Deal J.A., Reed N.S. (2023). Hearing loss and dementia prevalence in older adults in the US. JAMA..

[10] Jafari Z., Kolb B.E., Mohajerani M.H. (2019). Age-related hearing loss and tinnitus, dementia risk, and auditory amplification outcomes. Ageing Res Rev..

[11] Guan L., Liu Q., Chen D., Chen C., Wang Z. (2022). Hearing loss, depression, and medical service utilization among older adults: evidence from China. Public Health..

[12] Simpson A.N., Matthews L.J., Cassarly C., Dubno J.R. (2019). Time from hearing aid candidacy to hearing aid adoption: a longitudinal cohort study. Ear Hear..

[13] H-YH Lin, Willink A., Jilla A.M. (2021). Healthcare-seeking behaviors among medicare beneficiaries by functional hearing status. J Aging Health..

[14] McConnaughy E.A., Prochaska J.O., Velicer W.F. (1983). Stages of change in psychotherapy: measurement and sample profiles. Psychotherapy: Theory Res Pract..

[15] Saunders G.H., Frederick M.T., Silverman S., Papesh M. (2013). Application of the health belief model: development of the hearing beliefs questionnaire (HBQ) and its associations with hearing health behaviors. Int J Audiol..

[16] Laplante-Lévesque A., Hickson L., Worrall L. (2013). Stages of change in adults with acquired hearing impairment seeking help for the first time: application of the transtheoretical model in audiologic rehabilitation. Ear Hear..

[17] Brice S., Almond H. (2023). Behavior change in chronic health: reviewing what we know, what is happening, and what is next for Hearing Loss. Int J Environ Res Public Health..

[18] Arnold M., Small B.J., Hyer K. (2019). Development of a hearing help-seeking questionnaire based on the theory of planned behavior. Int J Audiol..

[19] Hickson L., Meyer C., Lovelock K., Lampert M., Khan A. (2014). Factors associated with success with hearing aids in older adults. Int J Audiol..

[20] Folstein M.F., Folstein S.E., McHugh P.R. (1975). “Mini-mental state”: a practical method for grading the cognitive state of patients for the clinician. J Psychiatr Res..

[21] Henseler J., Ringle C.M., Sarstedt M. (2015). A new criterion for assessing discriminant validity in variance-based structural equation modeling. J Acad Mark Sci..

[22] Fornell C., Larcker D.F. (1981). Evaluating structural equation models with unobservable variables and measurement error. J Mark Res..

[23] He P., Wen X., Hu X. (2018). Hearing aid acquisition in Chinese older adults with hearing loss. Am J Public Health..

[24] Meister H., Grugel L., Meis M. (2014). Intention to use hearing aids: a survey based on the theory of planned behavior. Patient Prefer Adherence.

